# Prevalence of Bertolotti’s Syndrome in Lumbosacral Surgery Procedures

**DOI:** 10.7759/cureus.26341

**Published:** 2022-06-26

**Authors:** Ahmed Ashour, Ahmed Hassan, Mohamed Aly, Mahmoud AM Nafady

**Affiliations:** 1 Trauma and Orthopaedics, Queen Elizabeth Hospital Birmingham, Birmingham, GBR; 2 Orthopaedics, Queen Elizabeth Hospital Birmingham, Birmingham, GBR; 3 Trauma and Orthopaedics, East Kent Hospitals University NHS Foundation Trust, Ashford, GBR; 4 Orthopaedics, El Hadra University Hospital, Alexandria, EGY

**Keywords:** micronucleotomy, plif, degeneration, lumbosacral surgery, bertolotti’s syndrome, lstv

## Abstract

Introduction

Bertolotti’s syndrome (BS) describes the relationship between low back pain (LBP) and lumbosacral transitional vertebra (LSTV). It is a factor that is sometimes overlooked when it comes to evaluating and treating LBP.Because of the different diagnostic modalities and criteria used in the research, the LSTV incidence in the general population varies greatly, and hence the link between LSTV and LBP remains contentious.^ ^Some researchers found no link between low back pain and LSTV. As a result, the management of BS remains controversial and multiple treatments have been suggested, including locally injected steroid and various surgical approaches.

Methods

This retrospective cohort study included a total of 288 patients who underwent lumbosacral surgical procedures for disc prolapse, lumbar canal stenosis, spondylolithesis and post-laminectomy syndrome during the period between January 2016 and May 2020. Trauma, tumours and scoliotic patients were excluded. All data were collected from the departmental database. All cases were done by the same surgical team at El Hadra University Hospital Spine Unit, Egypt. The patients were divided into two groups. Group A consisted of 133 patients in whom LSTV was detected by radiologic findings. In contrast, Group B consisted of 155 patients in whom LSTV was not detected.

Results

In our study, the overall prevalence of LSTV among 288 patients who underwent lumbosacral surgical interventions was 46.2%. On comparing the incidence of surgical interventions between both groups, there was non-significant difference in most of surgical interventions. The incidence of L3-5 double-level posterior lumbar interbody fusion (PLIF) among LSTV patients was 16.5% compared to 4.61% in the other group. The incidence of L4-S1 double-level PLIF among LSTV patients was 15.04% compared to 7.24% in the other group.

Regarding adjacent segment pathology, the incidence of lumbar canal stenosis and degenerative spondylolithesis was higher in the LSTV group (20.3% and 11.3%, respectively) compared to the non-LSTV group (9.7% and 5.2%, respectively). The incidence of disc prolapse was lower in the LSTV group (56.39%) compared to the non-LSTV group (71.0%). There was a non-significant difference between the incidence of lytic spondylolithesis and postlaminectomy syndrome between both groups.

Conclusion

The overall prevalence of LSTV among all cases who underwent lumbosacral surgical procedures at the El Hadra University Hospital was 46.2%. The incidence of lumbar canal stenosis and degenerative spondylolithesis was higher in the LSTV group compared to the non-LSTV group. However, the incidence of disc prolapse was lower in the LSTV group compared to the non-LSTV group. The incidence of disc prolapse and degenerative spondylolithesis at the L4-5 level was higher in the LSTV group compared to the non-LSTV group. In contrast, the incidence at L5-S1 was lower in the LSTV group compared to the non-LSTV group. Hence, LSTV is considered a risk factor for disc degenerative changes at the level above the transitional vertebra level.

## Introduction

Lumbosacral transitional vertebra (LSTV) is a congenital spinal malformation characterized by sacralization of the caudal lumbar vertebra or lumbarization of the most cephalad sacral segment of the spine. Mario Bertolotti, in 1917, was the first to suggest a link between low back discomfort and congenital structural anomalies in the last lumbar vertebra, which he called "sacral assimilation of the lumbar vertebrae" [1]. Bertolotti's syndrome defines the association of a transverse mega-apophysis in the lowest lumbar vertebra with a transitional characteristic linked with low back pain (LBP) [2].

The aberrant articulation itself, early degeneration and instability of the adjacent level proximal to the transitional vertebrae, the contralateral facet joint (in unilateral LSTV), and compression of nerve root from transverse process hypertrophy can all cause symptoms. Symptoms related to each of the mentioned causes are treated differently, necessitating the use of reliable procedures not only to diagnose LSTV but also to define the site and type of the pathology caused by the transitional segment [3,4].

## Materials and methods

This retrospective cohort study was approved by Institutional Review Board of the El Hadra University Hospital, Egypt. A total of 288 patients who underwent lumbosacral surgical procedures during the last five years between January 2016 and May 2020 were considered for inclusion in this study. Data were collected from the departmental database. All cases were managed by the same surgical team at the El Hadra University Hospital Spine Unit.

Inclusion criteria included patients who had single-, double-, and three-level posterior lumbar interbody fusion (PLIF), laminectomy, micronucleotomy and foraminectomy. Trauma patients, patients with spinal tumours and scoliosis patients (adult idiopathic scoliosis) were excluded. The patients were divided into two groups. Group A consisted of 133 patients in whom LSTV was detected by radiologic findings. In contrast, Group B had a total of 155 patients in whom LSTV was not detected.

Radiologic diagnosis of LSTV

Preoperatively, plain radiographs and magnetic resonance imaging (MRI) were done for all patients.

X-ray

While LSTV can be identified by any imaging modality, Ferguson radiographs have been described to provide the best views to identify them (anteroposterior [AP] radiographs angled at 30° cranially). Also, AP, oblique and lateral views are important [5].

Computed Tomography

Computed tomography (CT) is considered the best imaging technique for the identification of LSTV. These abnormalities are usually identified incidentally as CT is not usually indicated alone to identify LSTV, because of its radiation concerns. Therefore, it is the preferred imaging modality to evaluate patients with non-traumatic LBP [5].

Magnetic Resonance Imaging

Because of its greater tissue differentiation within and adjacent to the spine, MRI is used more frequently. Due to factors such as limited imaging of the thoracolumbar vertebral junction, difficult identification of the most caudal rib-bearing vertebral body and hard differentiation between the enlarged lumbar transverse processes and thoracic hypoplastic ribs, the numbering and classification of LSTV on MRI are the most difficult. These factors pose a dilemma for radiologists as they have to read an MRI scan of lumbar spine in isolation without using other imaging modalities such as spine radiographs to help identify LSTV correctly and enumerate them [5].

LSTV was classified using plain radiographs, and using the classification outlined by Castellvi et al. [6,7]. Type I has a large transverse process. Type II is defined by the presence of a diarthrodial joint between the transverse process of L5 and the ala of sacrum (a, unilateral; b, bilateral). In Type III, there is a true osseous union between the L5 transverse process and the ala of sacrum (a, unilateral; b, bilateral). Type IV is defined by the presence of both Type II on one side and Type III on the contralateral side.

Method of treatment

All patients in this study were subjected to laminectomy, micronucleotomy and PLIF.

Statistical analysis

After data entry in a specific sheet using Microsoft Excel XP (Microsoft Corp., Redmond, Washington), and following revision and correction of any data entry errors, the data were transferred to SPSS Statistics, version 24 (IBM Corp., Armonk, NY) and the following statistical analyses were performed: (1) arithmetic mean; (2) standard deviation (SD); (3) t-test to compare the means of two groups; (4) chi-square (χ^2^) for the comparison between the distribution of patients according to different items of the study.

## Results

The overall incidence of LSTV in cases that underwent lumbosacral surgery procedures was 46.2% (Table 1).

**Table 1:** Incidence of LSTV in lumbosacral surgery procedures LSTV, lumbosacral transitional vertebra; No., number

	No.	%
LSTV	133	46.2
No LSTV	155	53.8
Total	288	100.0

Comparison of the incidence of surgical interventions in the two groups

The incidence of L4-5 single PLIF and L5-S1 single PLIF was higher in the non-LSTV group (42.11% and 17.76%, respectively) compared to the LSTV group (32.33% and 15.04%, respectively). The incidence of L3-5 double PLIF and L4-S1 double PLIF was higher in the LSTV group (16.54% and 15.04%, respectively) compared to the non-LSTV group (4.61% and 7.24%, respectively).

**Figure 1:**
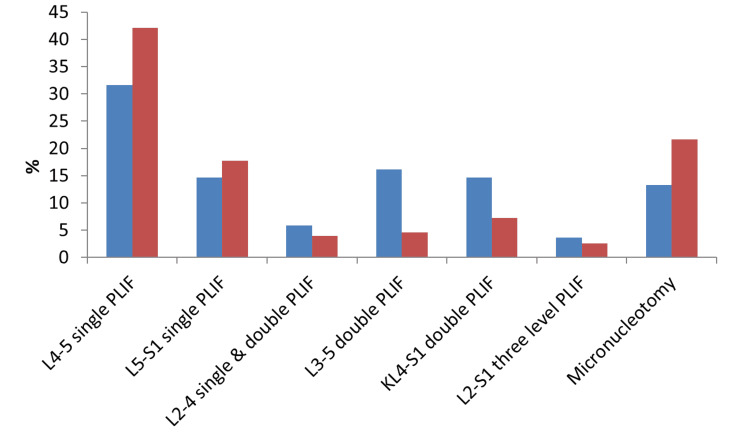
Comparison between the incidence of surgical interventions in the two groups LSTV, lumbosacral transitional vertebra; PLIF, posterior lumbar interbody fusion

On comparing both groups, the results were statistically non-significant (Table 2; Figure 1).

**Table 2:** Comparison between the incidence of surgical interventions in the two groups LSTV, lumbosacral transitional vertebra; PLIF, posterior lumbar interbody fusion; No., number

	Group A (LSTV), n=133		Group B (no LSTV), n=155		Total, n=288		χ^2^	P value
	No.	%	No.	%	No.	%		
L4-5 single PLIF	43	32.33	64	42.11	107	37.2	1.05	0.103
L5-S1 single PLIF	20	15.04	27	17.76	47	16.3	0.23	0.89
L2-4 single and double PLIF	8	6.02	6	3.95	14	4.9	0.41	0.698
L3-5 double PLIF	22	16.54	7	4.61	29	10.1	1.07	0.145
L4-S1 double PLIF	20	15.04	11	7.24	31	10.8	1.1	0.105
L2-S1 three-level PLIF	5	3.76	4	2.63	9	3.1	0.27	0.88
Micronucleotomy	18	13.53	33	21.71	51	17.7	1.25	0.144

Comparison of the incidence of adjacent segment pathology between the two groups

**Figure 2:**
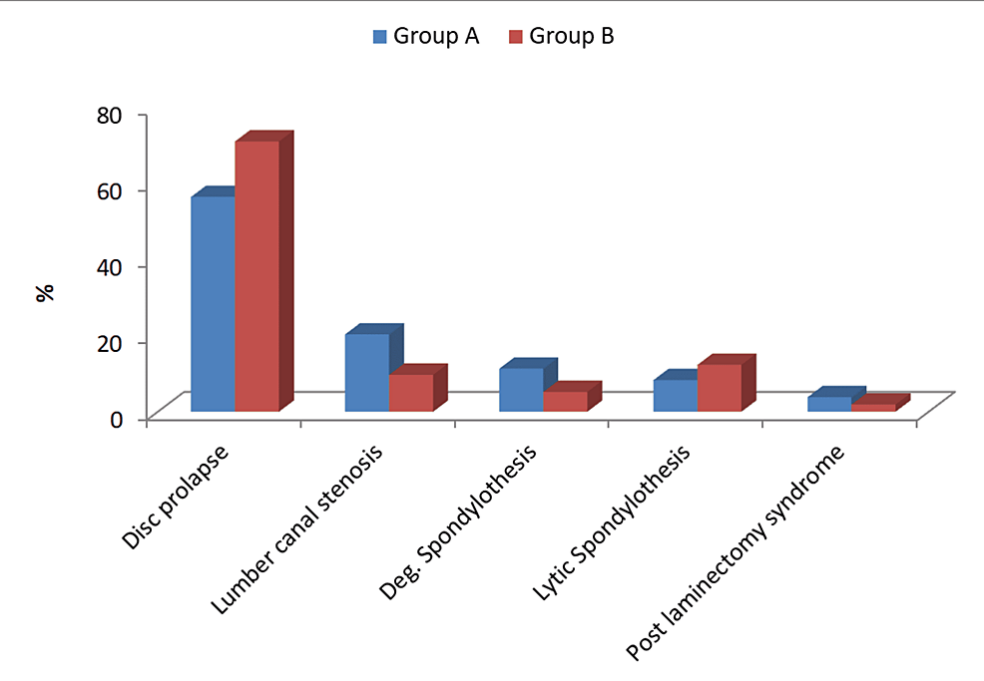
Comparison of the incidence of adjacent segment pathology between the two groups Deg., degenerative

Immediate postoperative X-rays were used for comparison. The incidence of disc prolapsed was higher in Group B (71%) compared to Group A (56.4%); the result was statistically significant. The incidence of lumbar canal stenosis was higher in Group A (20.3%) compared to Group B (9.7%), and the result was statistically significant. The incidence of degenerative spondylothesis was higher in Group A (11.3%) compared to Group B (5.2%); here also, the result was statistically significant (Table 3; Figure 2).

**Table 3:** Comparison of the incidence of adjacent segment pathology between the two groups LSTV, lumbosacral transitional vertebra; No., number

	Group A (LSTV), n=133		Group B (no LSTV), n=155		χ^2^	P value
	No.	%	No.	%		
Disc prolapse	75	56.4	110	71.0	2.14	0.021
Lumber canal stenosis	27	20.3	15	9.7	2.07	0.033
Degenerative spondylothesis	15	11.3	8	5.2	1.98	0.041
Lytic spondylothesis	11	8.3	19	12.3	1.42	0.098
Postlaminectomy syndrome	5	3.8	3	1.9	1.33	0.103

Comparison between the incidence of location of disc prolapse between both groups

**Figure 3:**
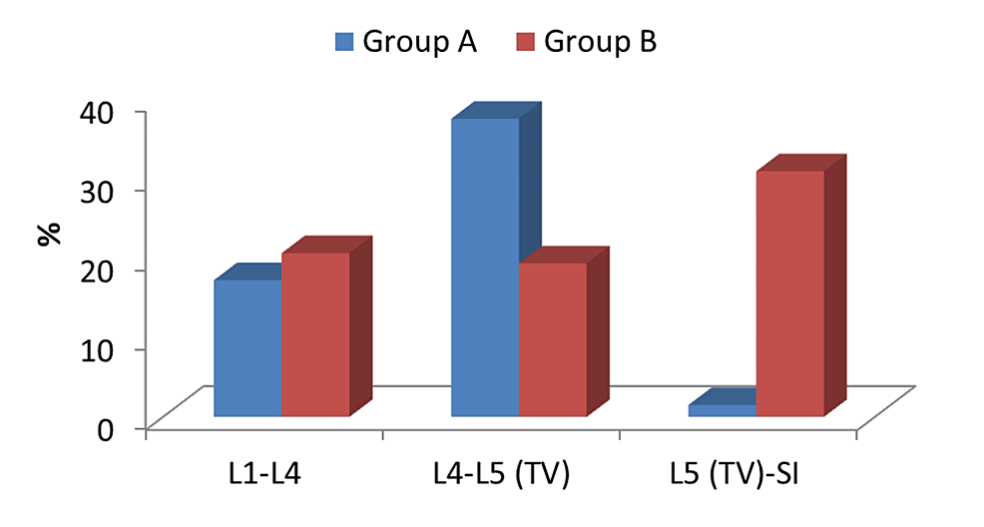
Comparison between the two studied groups regarding the location of disc prolapsed L, lumbar; TV, transitional vertebra; S, sacral

The incidence of disc prolapse at L4-5 was higher in the LSTV group (37.5%) compared to the non-LSTV group (19.3%). The incidence of disc prolapse at L5-S1 was lower in the LSTV group (1.5%) compared to the non-LSTV group (30.9%). On comparing both groups regarding both levels, the results were highly significant statistically (Table 4; Figure 3).

**Table 4:** Comparison between the two groups regarding the location of disc prolapse LSTV, lumbosacral transitional vertebra; S, sacral; No., number

Level	Group A (LSTV), n=133		Group B (no LSTV), n=155		χ^2^	P value
	No.	%	No.	%		
L1-L4	23	17.2	32	20.6	1.85	0.064
L4-L5 (TV)	50	37.5	30	19.3	3.98	0.003
L5 (TV)-S1	2	1.5	48	30.9	12.5	0.001

Comparison between the incidence of the location of degenerative spondylolithesis between both groups

**Figure 4:**
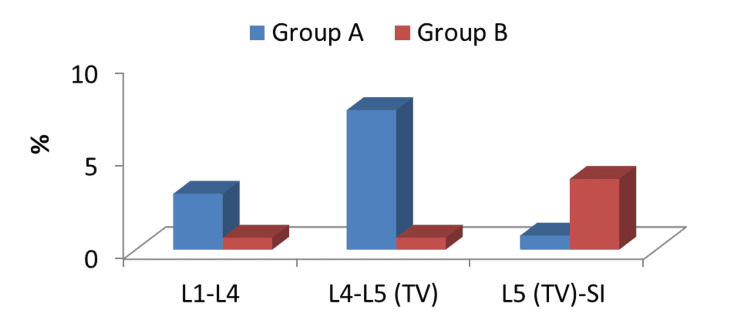
Comparison between the two studied groups regarding the location of degenerative spondylolithesis L, lumbar; TV, transitional vertebra; S, sacral

The incidence of degenerative spondylolithesis at L4-5 was higher in the LSTV group (7.5%) compared to the non-LSTV group (0.64%). The incidence of degenerative spondylolithesis at L5-S1 was lower in the LSTV group (0.75%) compared to the non-LSTV group (3.8%). On comparing both groups regarding both levels, the results were highly significant statistically (Table 5; Figure 4).

**Table 5:** Comparison between the two groups regarding the location of degenerative spondylolithesis LSTV, lumbosacral transitional vertebra; S, sacral; No., number

Level	Group A (LSTV), n=133		Group B (no LSTV), n=155		χ^2^	P value
	No.	%	No.	%		
L1-L4	4	3.0	1	0.64	1.21	0.107
L4-L5 (TV)	10	7.5	1	0.64	5.21	0.001
L5 (TV)-S1	1	0.75	6	3.8	2.65	0.013

Comparison between the incidence of location of lumbar canal stenosis between both groups

**Figure 5:**
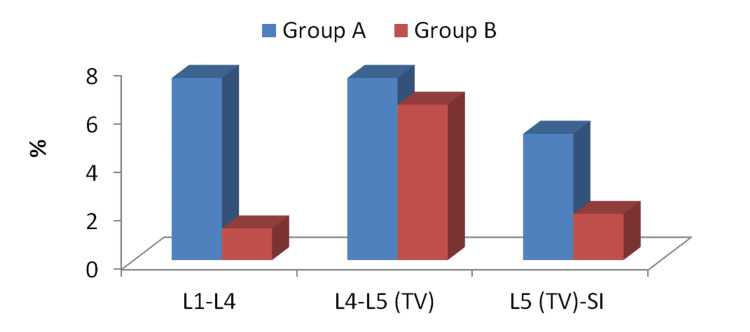
Comparison between the two studied groups regarding the location of spinal canal stenosis L, lumbar; TV, transitional vertebra; S, sacral

The comparison between the incidence of location of lumbar canal stenosis at all levels between both groups was non-significant statistically (Table 6; Figure 5).

**Table 6:** Comparison between the two groups regarding the location of spinal canal stenosis LSTV, lumbosacral transitional vertebra; S, sacral

Level	Group A (LSTV), n=133		Group B (no LSTV), n=155		χ^2^	P value
	No.	%	No.	%		
L1-L4	10	7.5	2	1.3	1.02	0.254
L4-L5 (TV)	10	7.5	10	6.4	0.112	0.85
L5 (TV)-S1	7	5.2	3	1.9	1.03	0.141

## Discussion

The relationship between LBP and LSTV, termed “Bertolotti’s syndrome”, was first mentioned by Bertolotti in 1917 [1]. Since then, many studies have been conducted on this subject giving different, controversial results. Although some did not show any relationship between LSTV and LBP, many of them supported this correlation.

Greater than expected numbers of LSTV cases have been found in patients being imaged for LBP or undergoing surgery for disc pathology. Multiple studies showed a higher incidence of disc pathology superjacent to LSTV. Luoma et al. found an increased incidence of upper disc early degeneration in young patients [8]. However, these changes were obscured by age-related degeneration in the middle-aged group. Epstein et al. reported an increase in disc prolapse in adolescent patients with spinal anomalies, including LSTV [9].

Extraforaminal stenosis causing nerve root entrapment and radiculopathy was described in patients with LSTV. Best imaged on coronal MRI, the nerve root is seen compressed between the LSTV transverse process and adjacent ala of sacrum [10]. Additionally, patients with symptoms of nerve root entrapment and LSTV are more likely to have disc herniation at the level above LSTV compared to those without LSTV. Furthermore, in the absence of spondylolisthesis, spinal canal stenosis is more likely to be at the disc above the LSTV. Assessing symptoms of nerve root entrapment in patients with LSTV may be complicated because there might be associated variations of lumbosacral myotomes. When a sacralized L5 vertebra is present, the nerve root of L4 provides the usual function of the L5 nerve root [11]. Similarly, when there is a lumbarized S1 vertebra, the S1 nerve root acts as the L5 nerve root. McCulloch and Waddell stated that the functional nerve root of L5 usually arises from the mobile lowest level of lumbosacral spine [12].

In our study, the overall prevalence of LSTV among 288 patients who underwent lumbosacral surgical interventions was 46.2%. On comparing the incidence of surgical interventions between both groups, a non-significant difference was found in most of surgical interventions. The incidence of L3-5 double-level PLIF among LSTV patients was 16.5% compared to 4.61% in the other group. The incidence of L4-S1 double-level PLIF among LSTV patients was 15.04% compared to 7.24% in the other group.

Regarding adjacent segment pathology, the incidence of lumbar canal stenosis and degenerative spondylolithesis was higher in the LSTV group (20.3% and 11.3%, respectively) compared to the non-LSTV group (9.7% and 5.2%, respectively). The incidence of disc prolapse was lower in the LSTV group (56.39%) compared to the non-LSTV group (71.0%). There was a non-significant difference between the incidence of lytic spondylolithesis and postlaminectomy syndrome between both groups.

Vergauwen et al. found that apart from nerve root canal stenosis, there was a non-significant difference in the incidence of structural problems between both groups including disc protrusion and/or extrusion, spinal canal stenosis, disc degeneration and facet degeneration [13]. de Bruin et al. concluded that LSTV is of low clinical significance in the early diagnosis of axial spondyloarthritis [14]. There was a non-significant difference between cases with and without LSTV in regard to the prevalence of axial spondyloarthritis, back pain and spinal mobility, and a bone marrow edema-like (BME) change in the pseudoarticulation does not reach sacroiliac joints.

In this study, we did a comparison between the incidence of the site of adjacent segment pathology between two groups; the incidence of disc prolapse and degenerative spondylolithesis at the L4-5 level was higher in the LSTV group (37.5% and 7.5%, respectively) compared to the non-LSTV group (19.3% and 0.64%, respectively). However, the incidence of both pathologies at L5-S1 was lower in LSTV patients (1.5% and 0.75%, respectively) compared to non-LSTV patients (30.9% and 3.8%, respectively). There was a non-significant difference in the incidence of the site of lumbar canal stenosis between both groups. The reason for these results remains unclear.

These findings are similar to a previous study done by Elster who reported that disc bulge and/or herniation is around nine times more common at disc space immediately above the LSTV than at any other level [15]. From a biomechanical point of view, this higher incidence of degeneration could be caused by the relative hypermobility of the vertebra above the LSTV. This is similar to the hypermobility reported in spinal segments adjacent to block vertebrae. The disc space immediately below the LSTV is usually vestigial and has a residual nuclear material and rarely has a pathological degeneration or alteration.

Ahn et al. did a retrospective study that included 398 patients who were followed up for two years following micronucleotomy for L4/5 disc herniation (disc superior to the LSTV) [16]. Postoperatively, the visual analog scale (VAS) scores of the back and leg decreased markedly in both groups. However, at 12 and 24 months postoperatively, the intensity of back pain worsened in LSTV group. Regarding secondary outcome measures, both 12-item Short-Form Health Survey and Oswestry Disability Index scores deteriorated at 12 and 24 months after surgery in the LSTV group. Two cases of recurrence of disc herniation (6.5%) were observed and required reoperation.

Peterson et al. in a study of 353 subjects with low back pain concluded that there was no difference in the levels of pain or disability between both groups according to any of the pain scores or Revised Oswestry Disability subscales [17]. Older patients demonstrated significantly more pain and disability (P=0.039, 0.002, respectively) than younger patients.

Our study has many strengths. Firstly, this is the first study, to our knowledge, to estimate the prevalence of LSTV among cases who underwent lumbosacral surgery procedures. Other studies mainly reviewed the incidence of Bertolotti’s syndrome in the general population and its relation to back pain. Secondly, this study included 288 cases of lumbosacral surgical interventions, which is a big number. Thirdly, the same surgical team operated on all cases and in the same hospital.

It is recommended that a larger sample size should be included in further studies. Long-term studies will be needed to assess clinical outcomes. Future studies are necessary to compare clinical outcomes between both groups. Further scores and questionnaires can be used to have accurate results.

## Conclusions

Our study concluded that the overall incidence of LSTV among all cases who underwent lumbosacral surgical procedures at the El Hadra University Hospital is 46.2%. LSTV is considered a risk factor for disc degenerative changes at the level above the transitional vertebra level. The incidence of lumbar canal stenosis and degenerative spondylolithesis was higher in the LSTV group compared to the non-LSTV group. However, the incidence of disc prolapse was lower in LSTV compared to non-LSTV patients. The incidence of disc prolapse and degenerative spondylolithesis at the L4-5 level was higher in the LSTV group compared to the non-LSTV group. In contrast, the incidence at L5-S1 was lower in LSTV compared to non-LSTV patients.
